# Genome Sequences and Photosynthesis Gene Cluster Composition of a Freshwater Aerobic Anoxygenic Phototroph, *Sandarakinorhabdus* sp. Strain AAP62, Isolated from the Shahu Lake in Ningxia, China

**DOI:** 10.1128/genomeA.00034-13

**Published:** 2013-02-28

**Authors:** Yonghui Zeng, Fuying Feng, Yapeng Liu, Yunxu Li, Michal Koblížek

**Affiliations:** a Institute of Microbiology CAS, Opatovický mlýn, Třeboň, Czech Republic; b Guangdong Provincial Key Laboratory of Pathogenic Biology and Epidemiology for Aquatic Economic Animals, Guangdong Ocean University, Zhanjiang, China; c Institute for Applied & Environmental Microbiology, Life Sciences College, Inner Mongolia Agriculture University, Huhhot, China; d Faculty of Science, University of South Bohemia, Branišovská, České Budějovice, Czech Republic

## Abstract

We report the first genome sequence from the recently established alpha-4 proteobacterial genus *Sandarakinorhabdus*. The genome of the *Sandarakinorhabdus* sp. strain AAP62 contains a photosynthesis gene cluster carrying major genes for bacterial reaction centers. The presence of genes related to aerobic respiratory electron transport confirms the lifestyle of this organism as an aerobic anoxygenic photoheterotroph.

## GENOME ANNOUNCEMENT

Aerobic anoxygenic phototrophic (AAP) bacteria are photoheterotrophic organisms that utilize light energy as a supplement to their predominantly heterotrophic metabolism (1). This functional group represents a ubiquitous part of freshwater bacterioplankton (2–4) with representatives belonging to various subgroups of *Alpha*- and *Betaproteobacteria*. *Sandarakinorhabdus* is a recently established AAP genus within the alpha-4 subgroup of *Proteobacteria* which contains only one type species, *Sandarakinorhabdus limnophila* (5). To assess the metabolic potential and photosynthesis gene composition of *Sandarakinorhabdus* species, we sequenced the whole genome of *Sandarakinorhabdus* sp. strain AAP62 collected from the surface water of the Shahu Lake in the Ningxia Hui Autonomous Region, China, in November 2011. This orange-red strain was isolated by plating diluted lake water onto R2A agars and was found to produce bacteriochlorophyll *a* under aerobic conditions.

We extracted DNA using a genomic DNA purification kit (Tiangen Biotech Co., Beijing, China) and constructed a 300-bp DNA library for Illumina sequencing technology. An Illumina HiSeq 2000 platform was employed for sequencing. Approximately 3.53 Gb of raw data of 101-bp-long pair-end reads was generated. A total of 13,692,514 reads were quality controlled and trimmed on the Galaxy server (6) and then were *de novo* assembled using the Velvet program (ver. 1.2.08) (7). Various hash lengths between 31 and 91 were tested and an optimal assembly was achieved with a kmer size of 59. Contigs less than 300 bp long were removed from the final assembly. Genome coverage was ca. 160×. Annotation was performed with the RAST (8) and BASys (9) servers.

The draft genome consisted of 22 contigs with an N_50_ value of 257,928 bp and a total length of 3.13 Mb, containing 3,036 open reading frames (ORFs) and 46 tRNAs. The G+C content was 65.5%. A full-length 16S rRNA gene sequence of this isolate shows 99.4% identity to that of the type strain *Sandarakinorhabdus limnophila* so42. A 44,237-bp-long photosynthesis gene cluster (PGC) was located in a 549,987-bp-long contig with a gene organization of *crtK-bchP-pucC-bchGFNBHLM-puhABC-lhaA-*ORF*-acsF-puhE-*6×ORFs*-bchIDO-*5×ORFs*-crtCDF-bchCXYZ-pufBALMC*.

*Sandarakinorhabdus* sp. AAP62 lacks the genes of any autotrophic CO_2_ fixation pathway. The catabolic pathway contains genes encoding enzymes for the pentose phosphate pathway, the Entner-Doudoroff pathway, and the tricarboxylic acid cycle. The presence of genes encoding acetyl coenzyme A (acetyl-CoA) carboxylase, phosphoenolpyruvate carboxylase, and urea carboxylase indicates carboxylation activities. There are genes encoding carbon monoxide dehydrogenase and accessory proteins (*coxLGSM*). There are some sulfur cycling-related genes, e.g., the adenylyl-sulfate reductase and sulfite reductase genes. Nitrogenase and nitrite and nitrate reductase are absent. Various proteins that are involved in the aerobic respiratory electron transport chain were identified, including NADH dehydrogenase, cytochrome-*c* oxidase, succinate dehydrogenase, and hydrogen peroxidase. The presence of these proteins confirms the aerobic lifestyle of the *Sandarakinorhabdus* sp. AAP62. These findings indicate a great metabolic versatility in the members of this AAP genus and will provide more insights into the metabolism and ecology of AAP communities in freshwater environments.

### Nucleotide sequence accession numbers.

This whole-genome shotgun project has been deposited at DDBJ/EMBL/GenBank under the accession number ANFY00000000. The version described in this paper is the first version, ANFY01000000.
